# Invasive Intracranial Aspergillosis in an Immunocompetent Patient

**DOI:** 10.1590/0037-8682-0106-2024

**Published:** 2024-05-06

**Authors:** Kemal Buğra Memiş, Elif Tan, Sonay Aydın

**Affiliations:** 1 Erzincan University, School of Medicine, Department of Radiology, Erzincan, Turkey.

**FIGURE 2:**
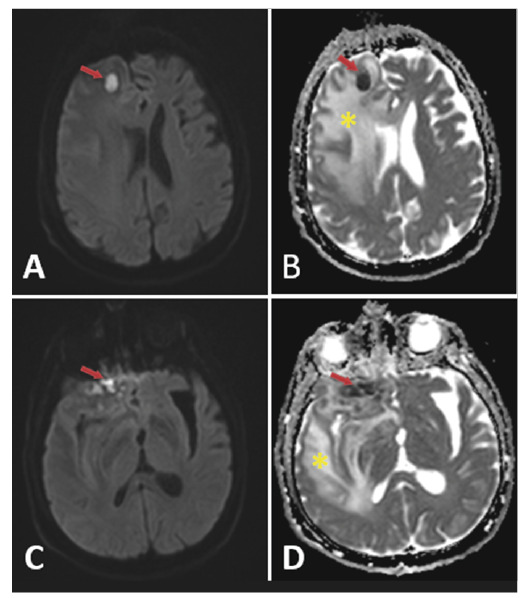
A 68-year-old male patient with intracranial aspergillosis. An abscess **(Red arrows)** exhibiting diffusion restriction is observed in the right frontal lobe denoted by hyperintense signals on diffusion-weighted images **(A,C)** and hypointense signals on apparent diffusion coefficient maps **(B,D)**. Furthermore, concurrent vasogenic edema **(yellow asterisk)** is observed in the right cerebral hemisphere.

A 68-year-old immunocompetent male patient with a history of sinonasal surgery and fungal sinusitis was hospitalized with headache, right eye pain, and vision loss. Computed tomography (CT) of the patient’s brain revealed multiple well-defined hypodense lesions with a hyperdense rim and areas of widespread vasogenic edema (Figure 1). Significant diffussion restriction was observed in these lesions (Figure 2). Additionally, there was a 5-mm left-to-right shift in the midline structures. Following CT and magnetic resonance imaging, we confirmed the diagnosis of aspergillosis using a stereotactic biopsy. Antifungal medications were subsequently initiated. The patient declined the recommended surgery due to concerns about potential risks. The development of this infection without known immune suppression suggests that it may be secondary to intracranial interventions.

**FIGURE:**
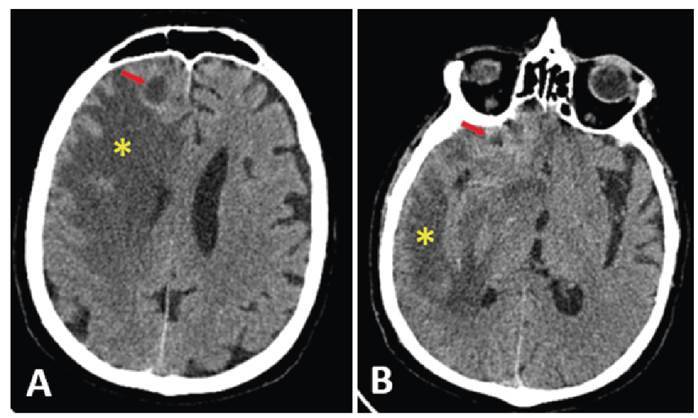
**1:** A 68-year-old male patient with intracranial aspergillosis. **A-B:** The axial non-contrast brain CT shows centrally hypodense lesions with peripheral hyperdense areas, compatible with an abscess **(red arrows)**, in the anterior part of the right frontal lobe. Additionally, there is extensive vasogenic edema in the surrounding brain

Cerebral involvement is observed in 10-20% of invasive aspergillosis cases and is associated with a high mortality rate (45-94%)^1^ ^,^ ^2^. They may occur as solitary or multiple cerebral abscesses, meningitis, epidural abscesses, or subdural hemorrhage^2^ ^,^ ^3^. It usually occurs because of hematogenous spread; however, it rarely occurs through a direct extension of paranasal sinuses^4^. The diagnosis was established using histopathology, direct microscopic examination, culture, serology, and imaging^3^ ^,^ ^4^. Patients who have previously undergone an intracranial intervention for a fungal infection should be carefully monitored during the postoperative period, and precautions should be taken to prevent the potential development of other fungal infections.
